# RETRACTION: Evaluating the Therapeutic and Reconstructive Efficacy of Flap Transplantation Techniques in Managing Nasal Tissue Deficiency Resulting From Post‐Rhinoplasty Surgical Infections

**DOI:** 10.1111/iwj.70611

**Published:** 2025-04-21

## Abstract

**RETRACTION:** songL. and LiuX. , “Evaluating the Therapeutic and Reconstructive Efficacy of Flap Transplantation Techniques in Managing Nasal Tissue Deficiency Resulting From Post‐Rhinoplasty Surgical Infections,” International Wound Journal 21, no. 2 (2024): e14566, 10.1111/iwj.14566.38379268 PMC10809024

The above article, published online on 25 January 2024, in Wiley Online Library (http://onlinelibrary.wiley.com/), has been retracted by agreement between the journal Editor in Chief, Professor Keith Harding; and John Wiley & Sons Ltd. Following an investigation by the publisher, all parties have concluded that this article was accepted solely on the basis of a compromised peer review process. The editors have therefore decided to retract the article. The authors did not respond to our notice regarding the retraction.

**RETRACTION:** L. song and X. Liu , “Evaluating the Therapeutic and Reconstructive Efficacy of Flap Transplantation Techniques in Managing Nasal Tissue Deficiency Resulting From Post‐Rhinoplasty Surgical Infections,” International Wound Journal 21, no. 2 (2024): e14566, 10.1111/iwj.14566.38379268 PMC10809024

The above article, published online on 25 January 2024, in Wiley Online Library (http://onlinelibrary.wiley.com/), has been retracted by agreement between the journal Editor in Chief, Professor Keith Harding; and John Wiley & Sons Ltd. Following an investigation by the publisher, all parties have concluded that this article was accepted solely on the basis of a compromised peer review process. The editors have therefore decided to retract the article. The authors did not respond to our notice regarding the retraction.

